# Development and validation of an eating behavior patient‐reported outcome measure in people living with obesity or overweight

**DOI:** 10.1002/oby.24299

**Published:** 2025-05-28

**Authors:** Donald M. Bushnell, Shirley Fung, Meryl Brod, Carl A. Roberts, Carel W. Le Roux, Oren Steen, Kathryn J. Lucas, Anita M. Hennige, Anastasia Uster

**Affiliations:** ^1^ PPD, Evidera, Thermo Fisher Scientific Wilmington North Carolina USA; ^2^ The Brod Group Mill Valley California USA; ^3^ Department of Psychology, Institute of Population Health University of Liverpool Liverpool UK; ^4^ St. Vincent's University Hospital and University College Dublin School of Medicine Dublin Ireland; ^5^ Private Practice Toronto Ontario Canada; ^6^ Diabetes and Endocrinology Consultants Morehead City North Carolina USA; ^7^ Boehringer Ingelheim International GmbH Biberach Germany; ^8^ Boehringer Ingelheim International GmbH Ingelheim am Rhein Germany; ^9^ Present address: Centers for Medicare & Medicaid Services Baltimore Maryland USA

## Abstract

**Objective:**

The objective of this study was to develop a measure of eating behaviors in adults living with obesity or overweight.

**Methods:**

Based on concept‐elicitation (*n* = 53) and cognitive‐debriefing (*n* = 15) studies, a draft eating behavior patient‐reported outcome (EB PRO) measure was developed. Its psychometric properties were established using data from a 46‐week clinical trial of an investigational antiobesity medication (*n* = 387).

**Results:**

The final EB PRO comprised 12 items across two domains (i.e., “Desire to Eat” and “Capacity to Resist”; 6 items each) and a Total Eating Behavior score (0–48; higher scores worse). The EB PRO demonstrated good test–retest reliability (intraclass correlation coefficients > 0.7). Confirmatory factor analysis showed a comparative fit index of 0.98 with good internal consistency (Cronbach α > 0.7). The EB PRO generally exhibited moderate‐to‐large correlations with the Patient Global Impression of Severity questionnaire and Three‐Factor Eating Questionnaire and weaker correlation with the 36‐Item Short‐Form Health Survey version 2 Physical Functioning scale. All EB PRO scores improved from baseline to week 46 (*p* < 0.0001), demonstrating sensitivity to change with therapy. Clinically meaningful thresholds were defined as eight‐ and four‐point changes in Total Eating Behavior and domain scores, respectively.

**Conclusions:**

The EB PRO measure is a promising tool for assessing eating behaviors in people with obesity or overweight.

**Study Importance**

**What is already known?**

- Altered eating behaviors may be associated with the disease of obesity.
- Patient‐reported outcome measures that were previously developed to evaluate eating behaviors in people with eating disorders may not capture relevant behaviors in people with obesity or overweight, may be cumbersome to employ, and may not meet regulatory requirements.

**What does this study add?**

- These studies developed a novel eating behavior patient‐reported outcome (EB PRO) measure for people with obesity or overweight.
- Psychometric properties of the measure were established in a series of qualitative and quantitative analyses, and the last used data from a phase 2 clinical trial of survodutide, an investigational dual agonist of the glucagon and glucagon‐like peptide‐1 receptors.

**How might these results change the direction of research or the focus of clinical practice?**

- The psychometric properties of the EB PRO measure suggest that it has several potential applications, including use in clinical research to evaluate disease pathophysiology and obesity interventions and to support regulatory approval of obesity interventions.
- The EB PRO measure could be used in clinical practice to help tailor dietary counseling by identifying individuals with drivers to eat and/or emotional or social eating, as well as to track on‐treatment changes in eating behaviors.

## INTRODUCTION

Obesity is associated with impaired eating behaviors [1]. Recently approved antiobesity medications (AOMs; more recently referred to as obesity‐management medications) based on the gastrointestinal hormone glucagon‐like peptide‐1 (GLP‐1), i.e., semaglutide (Wegovy) and tirzepatide (Zepbound/Mounjaro), have demonstrated substantially greater efficacy than older drugs for chronic weight management and thereby have been termed second‐generation AOMs [2]. These medications are typically indicated for individuals with a body mass index (BMI) ≥ 30 kg/m^2^ (obesity) or ≥27 kg/m^2^ (overweight) with at least one weight‐related comorbidity [3, 4]. Second‐generation AOMs have been associated with improvements in certain dimensions of eating behaviors such as better control of eating and reduced cravings [5, 6, 7, 8], as well as anecdotal reports of substantially reduced “food noise,” a colloquial term referring to persistent preoccupation with food and eating, which may be related to food‐cue reactivity [9].

Given the availability of these AOMs and similar second‐generation compounds in late‐stage clinical development for chronic weight management, together with the rapidly increasing prevalence of obesity and overweight [10], standardized methods for evaluating eating behaviors are of interest for both clinical management of this disease and investigation of novel therapies. However, many instruments used for assessing eating behaviors [11, 12, 13, 14, 15, 16, 17, 18, 19, 20, 21, 22, 23, 24] were developed for people with eating disorders rather than those with obesity or overweight, whereas other instruments developed to assess the impact of obesity focus on domains such as physical and emotional symptoms [25] rather than understanding its associated eating behaviors. Furthermore, these instruments may be cumbersome to employ and may not necessarily meet current regulatory requirements for patient‐reported outcome measures, including US Food and Drug Administration (FDA) guidance, such as evidence of content validity (including patient input in development), reliability, construct validity, and ability to detect change [26, 27, 28].

Therefore, we aimed to develop an eating behavior patient‐reported outcome (EB PRO) measure to specifically assess eating behaviors in people living with obesity or overweight. Our objective was to create a measure that comprehensively captures common eating behaviors that are prevalent in patients with obesity.

## METHODS

### Overall methodology

Development of the EB PRO measure involved qualitative and quantitative studies between April 2019 and June 2023. These studies included a targeted literature review, followed by concept‐elicitation research, and then development of a draft conceptual framework and initial item generation, cognitive debriefing, psychometric evaluation of the draft measure, and finalization of the conceptual framework and measure. At several stages, an expert panel (*n* = 11) comprising authors (Donald M. Bushnell, Shirley Fung, Meryl Brod, Carl A. Roberts, and Anastasia Uster) and other academic and clinical experts in chronic weight management, behavior, and clinical psychology, as well as patient advocates and patient‐reported outcome methodologists, met to discuss the findings and advise on further work. These studies were conducted according to the FDA guidance on patient‐reported outcome measures [26, 27, 28].

### Targeted literature review

In order to inform interviews with experts and the interview guide for the concept‐elicitation study, a targeted literature review of preexisting patient‐reported outcome instruments conducted in April 2019 searched the following: PubMed/MEDLINE, Embase, PsycINFO, abstracts from the previous two ObesityWeek and European Congress on Obesity conferences, ClinicalTrials.gov, US and European patient‐reported outcome labeling claims in obesity, and the US FDA's Clinical Outcome Assessment Compendium (2015 version).

### Concept elicitation

Concept elicitation comprised a cross‐sectional, qualitative study involving focus groups of US adults living with obesity or overweight. As an exploratory study, sample size was not formally prespecified, but 42 to 54 participants were targeted based on scientific and logistical considerations [29, 30]. Institutional review board approval (Advarra Inc.) of the study protocol and the informed consent form was obtained prior to participant recruitment. Individuals from Atlanta, Georgia; Chicago, Illinois; Los Angeles, California; and Philadelphia, Pennsylvania were recruited by a market research company from its in‐house database. Participants had to be aged ≥18 years, have BMI ≥ 27.0 kg/m^2^ (with ≥1 obesity‐related complication if BMI was 27.0–29.9 kg/m^2^), and be able to read, write, and speak English. Individuals were excluded if they reported on the eligibility form other serious medical conditions that, in the judgment of the screener, indicated that they had cognitive impairment or other medical conditions that would interfere with their ability to participate in an interview and/or complete the study procedures.

Participants provided written informed consent and then attended gender‐specific focus group sessions lasting ≤120 min that were moderated by trained staff using a semistructured interview guide, which included open‐ended questions designed to address participants' experience of eating behaviors and physical activities. All interviews were audio‐recorded, transcribed, and anonymized. Participants received $175 compensation.

Qualitative data from the focus groups were analyzed using grounded theory to construct a conceptual framework related to eating behaviors. The thematic coding method was employed, using qualitative data analysis software (ATLAS.ti version 8; https://atlasti.com/). Specifically, a coding dictionary was developed and refined iteratively to capture important concepts related to eating behaviors identified by focus group participants. Focus group transcripts were coded to preliminarily categorize participant responses and any themes that emerged. Coded text was extracted into a concept‐frequency grid to organize responses into thematic groups. A saturation table was constructed to ensure that eating behavior themes were comprehensive and reflected all relevant aspects of the measurement of the concept of interest from the population of interest.

### Item generation and development of draft conceptual framework

Eating behavior themes elicited in the focus groups were considered for inclusion in a draft conceptual framework. Preliminary items for the measure were developed in item‐generation meetings.

### Cognitive debriefing

Preliminary items were evaluated in cognitive debriefing, which involved a cross‐sectional, qualitative study with individual telephone interviews of adults living with obesity or overweight in eight US states, using the same institutional review board approval process, recruitment strategy, sample‐size considerations (with a target of 15 individuals), and eligibility criteria as the concept‐elicitation study.

Trained staff conducted one‐on‐one cognitive interviews lasting ≤90 min in which participants read and signed the informed consent form and then completed the draft EB PRO measure developed from the draft conceptual framework. Participants were then asked questions from a semistructured interview guide regarding their overall impression of the measure, clarity of instructions, length and recall period, relevance to disease, and their understanding of and suggestions for each item.

Three waves of interviews were iteratively conducted to refine the EB PRO measure. After each wave, the measure was modified to reflect participant perspectives from the previous wave. The interview guide was adapted to reflect these changes, and a modified version was administered during each subsequent wave. A saturation table was constructed to ensure that participant feedback on the measure was comprehensive and that no additional concerns about it were discovered. Interviews were audio‐recorded and transcribed. Participants received $150.

### Psychometric evaluation of draft measure and development of final conceptual framework and measure

The psychometric properties of the draft EB PRO measure were evaluated using data collected during a phase 2 clinical trial of survodutide (BI 456906) [31], a glucagon/GLP‐1 receptor dual agonist in development for chronic weight management in people living with obesity [32]. All analyses were prespecified and used pooled blinded data regardless of whether participants were on or off the study drug (survodutide or placebo). Participants with missing data for an item at a particular time point were excluded from analyses of that item at that time point and its corresponding domain. Significance level (α) was set at 0.05 (two‐sided) without adjustment for multiple analyses. SAS version 9.4 (SAS Institute Inc.) was used for statistical analyses, except when indicated otherwise.

#### Study sample and measures

In this randomized, double‐blind trial (ClinicalTrials.gov: NCT04667377), following a screening period of 1 to 12 weeks to check eligibility criteria, 387 people aged ≥18 to <75 years with BMI ≥ 27 kg/m^2^ without diabetes received once‐weekly subcutaneous injections of placebo or survodutide (0.6, 2.4, 3.6, or 4.8 mg) for 46 weeks, comprising an initial 20‐week dose‐escalation period followed by 26 weeks of maintenance treatment [31]. The trial protocol was approved by the relevant independent ethics committees or institutional review boards for the 43 participating sites in 12 countries. Individuals without depression or with mild‐to‐moderate depression were eligible (defined as Patient Health Questionnaire‐9 score < 15).

The draft EB PRO and the following measures were assessed at baseline, week 20, and week 46: the Patient Global Impression of Severity (PGIS) for Eating Behavior (a single‐item questionnaire designed to assess the severity of eating behavior attributes) [33]; the revised 18‐item version [12] of the Three‐Factor Eating Questionnaire (TFEQ‐R18V2; an instrument comprising three domains: uncontrolled eating, cognitive restraint, emotional eating) [11]; the 10‐item Physical Functioning scale (PF‐10), acute version, of the 36‐Item Short‐Form Health Survey version 2 (SF‐36v2) [34], comprising 10 physical function‐related items designed to measure functional health; and a 3‐day food diary in which participants recorded on paper all food and drinks for 3 consecutive days, including 2 days during the week and 1 during the weekend (on days typical of their eating pattern). Energy and macronutrient intake was calculated by trained personnel (dietitian or equivalent staff) at study sites based on the description of consumed food/beverages and interview with the participant. The EB PRO and PGIS were also assessed at screening (the assessment prior to the baseline assessment). The Patient Global Impression of Change (PGIC), a one‐item questionnaire designed to compare overall eating behavior during and after treatment, was assessed at weeks 20 and 46 [35].

#### Factor analyses

Exploratory factor analyses were used to examine the underlying structure among the draft EB PRO items, using data from the screening and baseline assessments. Factors were derived based on evaluation of eigenvalues, scree plot, and interpretation of simple structure. Factor loadings > 0.40 were considered acceptable; rotation (i.e., varimax) was allowed. Confirmatory factor analysis using a structural equation modeling approach was conducted using week 20 and 46 data.

#### Item deletion/retention

Descriptive statistics for responses to the EB PRO items at screening, baseline, week 20, and week 46, as well as the factor analyses, were used to explore potential item reduction of the draft EB PRO measure and thereby determine the final conceptual framework and final measure. Spearman correlation coefficients were calculated for item‐to‐item correlations, item‐to‐total correlations, and correlations between scores for any factors identified. Decisions on item reduction were based on whether there was a high proportion of missing data (>50% of participants), high item‐to‐item correlation (>0.70), low item‐to‐total correlation (<0.40), or inadequate factor loading (<0.40 on any factor or >0.40 on >1 factor).

#### Reliability

Internal consistency reliability was evaluated using Cronbach α [36], with coefficients > 0.70 considered acceptable. Test–retest reliability was evaluated using screening and baseline data for stable participants, defined as those with no change in PGIS during that time period; intraclass correlation coefficients  > 0.70 were considered acceptable.

#### Validity

Convergent validity was evaluated by calculating Spearman rank correlation coefficients between EB PRO scores and the following measures: PGIS, SF‐36v2 PF‐10 (item 3: multi‐item scale score), TFEQ‐R18V2 (cognitive restraint scale, emotional eating scale, item 9, item 18), and the 3‐day food diary (daily energy intake). Correlations were defined as small (<0.3), moderate (0.3–0.7), or large (>0.7) in either positive or negative directions. It was hypothesized that correlations would be moderate to large with convergent constructs designed to report eating behaviors or diet and small with measures not specific to eating behaviors. Consequently, PGIS and TFEQ‐R18V2 were employed because they are widely used measures of eating behaviors, and the 3‐day food diary was used because it reported dietary intake in this study, whereas SF‐36v2 PF‐10 was employed because it does not measure eating behaviors. Known‐groups validity (hypothesizing that EB PRO scores would differ between PGIS and PGIC categories) was tested using ANOVA at baseline, week 20, and week 46, with EB PRO scores as the dependent variable and participant status/severity as the independent variable based on the following groups: PGIS (severity of hunger: not at all, a little, somewhat, very, or extremely), PGIC (much worse, a little worse, no change, a little better, or much better), and BMI category (25 to <30, 30 to <35, 35 to <40, or ≥40 kg/m^2^).

#### Sensitivity to change

ANOVA was used to evaluate differences in EB PRO scores between baseline and weeks 20 and 46 by changes in PGIS (improved, no change, or worsened) and PGIC (much worse, a little worse, no change, a little better, or much better). Effect size (mean change in score divided by SD of baseline score) and standard response mean (mean change divided by SD of change) were calculated.

#### Interpretation of meaningful change

Meaningful change in EB PRO scores was assessed with anchor‐ and distribution‐based approaches. The anchor‐based approach used PGIS, PGIC, and body weight as anchors. Spearman rank correlation coefficient was used to evaluate correlation between anchors and EB PRO scores/changes in scores at baseline and week 46. Coefficients ≥0.30 were considered to demonstrate at least moderate correlation. The distribution‐based approach calculated one‐half SD at baseline and SE of mean change at week 46.

## RESULTS

### Concept elicitation

#### Focus group characteristics

Six focus groups were conducted involving 53 individuals living with obesity or overweight. Overall mean age was 50.5 years (range 28–76), and mean BMI was 38.6 kg/m^2^; 49.1% were female (Table S1). Most participants identified as Black/African American (49%) or White (47%) and non‐Hispanic/Latino (86.8%). Most participants lived with others (69.8%) and had some form of employment (60.4%) and college education (62.3% completed or had some college). Self‐reported health of most participants (79.2%) was average or worse, and the majority reported at least one comorbidity, most commonly hypertension (56.6%), back and neck pain (45.3%), hypercholesterolemia (41.5%), and type 2 diabetes (34.0%).

#### Qualitative interviews

The most frequently reported eating patterns were “irregular meals” (55% of participants) and “eat until it's gone” (23%). The most common hunger‐related themes were “experience hunger once or twice a day” (43%), “do not experience hunger” (17%), and “overeats when experiencing real hunger” (17%). “Always hungry” was less common (6%). The most reported themes for eating triggers were “emotional eating” (57%), “social eating” (45%), “comfort eating” (32%), and “boredom eating” (30%). Regarding reward‐driven eating behaviors, the most common stimulants to strongly feeling pleasure and reward included highly palatable foods, such as “sweets” (60%), “carbohydrates” (38%), “salty” foods (32%), and “fat” foods (28%), and “strong food cues” (49%), including sight and smell. “Overeating related to highly palatable foods” was also commonly reported (36%). The most common themes for executive control (capacity to resist eating) were “reward overrides control/immediate gratification” (30%), “positive self‐control” (30%), and “a losing battle” (25%).

#### Draft conceptual framework

Based on participants' experiences, 35 eating behavior‐related themes were elicited; the most common (≥25% of participants) were irregular meal schedule (including frequent meals), snacks throughout the day, nighttime snacks, attraction to/preference for highly palatable foods, experiencing no real hunger or only experiencing it once or twice a day, strong food‐cue reactivity, reward overrides control, a losing battle, positive self‐control, emotional eating, social eating, cravings for specific foods, boredom eating, health condition‐related eating, and overeating for rewards.

Based on their frequency of endorsement by participants, potential to characterize the effects of therapies, and input from the expert panel, 13 themes were included in the draft conceptual framework, laid out across three domains: “Drivers to Eat,” “Capacity to Resist,” and “Eating Habits” (including “Homeostatic Hunger”). The latter domain was not as prominent as the first two but was included based on panel input.

### Cognitive debriefing

#### Participant characteristics

Fifteen people living with obesity or overweight participated in the three waves of cognitive interviews (*n* = 8, 4, and 3, respectively). Overall mean age was 49.7 years, mean BMI was 36.3 kg/m^2^, and 53.3% were female (Table S2). Participants identified as White (86.7%) or Black/African American (13.3%). Most (86.7%) lived with others and had either full‐ or part‐time employment (73.3%), whereas all had some college education. Most (73.3%) reported average or worse health. The most common obesity‐related complications were hypertension (46.7%), hypercholesterolemia (40.0%), back and neck pain (33.3%), and sleep apnea (33.3%).

#### Metrics for completing draft EB PRO measure

Mean time to complete the 23‐item draft EB PRO measure was 3.8 min, with 14 participants (93%) completing it in 3 to 5 min. All 15 (100%) indicated that the length was just right or reasonable and that the instructions were clear and easy to understand; none reported any difficulty completing it. All participants understood that the recall period was 7 days.

#### Qualitative results

**FIGURE 1:**
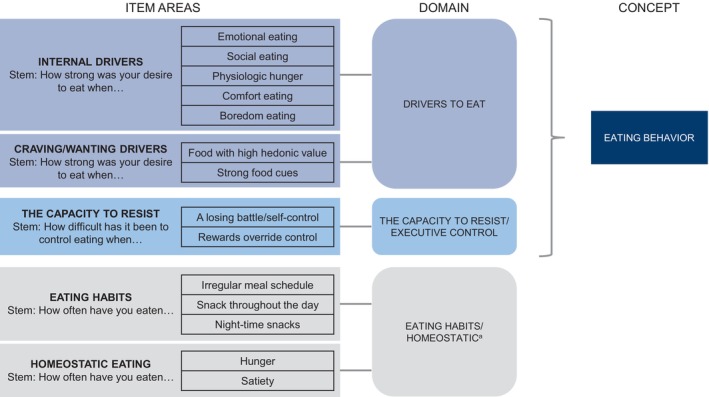
Draft conceptual framework for the EB PRO measure following cognitive‐debriefing interviews with people living with overweight or obesity. ^a^The “Eating Habits” domain did not emerge from the concept‐elicitation interviews but was included based on input from the expert panel. EB PRO, eating behavior patient‐reported outcome.

Across all waves, the majority of interviewees generally indicated that each domain (Drivers to Eat, Capacity to Resist, and Eating Habits/Homeostatic) captured all situations reflecting their desire to eat, ability to resist eating, and eating habits. Based on participant input in the interviews, several changes were made to the wording and order of items in the draft measure. From the wave 1 interviews, six changes were made to the wording; wave 2 interviews suggested that the changes were mostly effective in clarifying those items. Furthermore, findings from wave 2 led to new changes to four items. Wave 3 interviews indicated that all of the prior modifications were effective in reducing confusion and improving clarity; therefore, no further changes were made. Based on the three waves, two changes were made to the wording of the two scales of response options for intensity (“somewhat” to “moderately”) and frequency (“occasionally” to “sometimes”). The adjusted draft conceptual framework is shown in Figure 1.

### Psychometric evaluation of draft measure and development of final conceptual framework and measure

#### Study sample characteristics

In the survodutide phase 2 trial cohort employed for the psychometric analyses of the draft EB PRO measure, mean age was 49.1 years, mean BMI was 37.3 kg/m^2^, 68.2% were female, and 78.0% were White (Table 1). In the assessments of the draft measure at baseline, week 20, and week 46, only zero to three participants (0%–0.8%) had missing data at any time point for each item.

**TABLE 1:** Baseline characteristics of the psychometric evaluation study sample.

	Total (*N* = 387)
Age, mean (SD), y	49.1 (12.9)
Gender, *n* (%)	
Male	123 (31.8)
Female	264 (68.2)
Race, *n* (%)	
White	302 (78.0)
Black/African American	38 (9.8)
Asian	41 (10.6)
Native Hawaiian/Pacific Islander	2 (0.5)
American Indian/Alaska Native	1 (0.3)
Other	3 (0.8)
Ethnicity, *n* (%)	
Hispanic/Latino	18 (4.7)
Not Hispanic/Latino	369 (95.3)
BMI (kg/m^2^), *n* = 384, mean (SD)	37.3 (6.6)
BMI (kg/m^2^), *n* (%)	
27–29.9	37 (9.6)
≥30	347 (89.7)
Missing	3 (0.8)
Country of residence, *n* (%)	
United States	130 (33.6)
Canada	83 (21.4)
Australia	35 (9.0)
Sweden	25 (6.5)
South Korea	22 (5.7)
Netherlands	20 (5.2)
Poland	20 (5.2)
China	14 (3.6)
Germany	11 (2.8)
New Zealand	11 (2.8)
UK	9 (2.3)
Belgium	7 (1.8)

*Note*: Data are from a randomized, double‐blind, placebo‐controlled, phase 2 trial of survodutide, a glucagon/glucagon‐like peptide‐1 receptor dual agonist, in people living with obesity or overweight [31]. Data shown as mean (SD) for continuous variables and *n* (%) for categorical variables.

#### 23‐item draft EB PRO measure scores

Generally, scores for the 23 items in the draft measure were similar between screening and baseline but tended to improve at weeks 20 and 46, with the largest improvements from baseline to week 46 in items 9 (foods addictive), 10 (controlled by food), 13 (control portion size), 15 (avoid buying food resist), and 17 (difficulty resisting; Tables S3 and S4).

#### Item correlations

Interitem correlations at screening and baseline are shown in Table S5; the strongest associations were between items within the same domain, with correlations in the Eating Habits/Homeostatic domain much lower than in the other domains. Item‐to‐total correlations were generally moderate to strong (range: 0.20–0.84 at screening, 0.18–0.82 at baseline).

#### Factor analyses and item reduction

**FIGURE 2:**
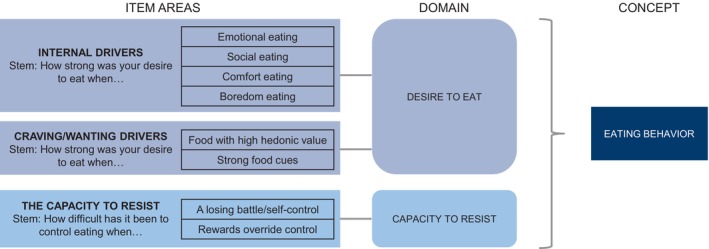
Final conceptual framework for the EB PRO measure. EB PRO, eating behavior patient‐reported outcome.

Exploratory factor analyses of the draft 23‐item EB PRO measure produced a final factor model with two domains of six items each. Based on these exploratory factor analyses, as well as item distributions and correlations, 11 items were removed from the final measure. Two items belonged to the Drivers to Eat domain (eating socially and eating when hungry), which was renamed to Desire to Eat, and three were within the Capacity to Resist domain (foods addictive, controlled by food, and difficulty resisting). The other six removed items were from the Eating Habits/Homeostatic domain (regular meals, snacked during day, snacked during evening/night, felt hungry, felt full, and satisfied by meal), which was thereby removed from the measure. Thus, the final conceptual framework for the EB PRO measure contains 12 items across two domains: Desire to Eat and Capacity to Resist (Figure 2). Each item is scored between 0 and 4, with domain scores ranging between 0 and 24 and Total Eating Behavior score between 0 and 48. Higher scores indicate stronger desire to eat and less capacity to resist.

Confirmatory factor analyses conducted on the 12 items in the final EB PRO using week 20 and 46 data showed adequate fit statistics (Table 2). The comparative fit index was 0.98, over the optimal threshold of 0.90. The root mean square error of approximation was 0.08, in the acceptable range of 0.05 to 0.08. These indices strongly suggest that the data fit the measurement model identified in the conceptual framework.

**TABLE 2:** Confirmatory factor analysis of the final EB PRO measure.

Domain/items	Factor loadings	
	Week 20 (*n* = 288)	Week 46 (*n* = 348)
Desire to Eat		
Item 1: Feeling emotional	0.820	0.736
Item 2: Eating with family and friends	0.679	0.715
Item 5: Indulge in comfort foods	0.780	0.756
Item 6: When you were bored	0.856	0.854
Item 7: Eat your favorite unhealthy foods	0.806	0.842
Item 8: Eat foods readily available	0.677	0.724
Capacity to Resist		
Item 11: Fight against eating habits	0.821	0.851
Item 12: Make good food choices	0.748	0.735
Item 13: Control portion size	0.858	0.846
Item 14: Control eating when hungry	0.870	0.898
Item 15: Avoid buying foods resist	0.808	0.843
Item 16: Avoid eating foods resist	0.864	0.883
Factor intercorrelations		
Capacity to Resist with Desire to Eat	0.864	0.898
Model fit statistics		
χ^2^ (degrees of freedom)	149.576 (53)	180.666 (53)
*p* value	0.0000	0.0000
Comparative fit index	0.981	0.985
Root mean square of approximation (90% CI)	0.080 (0.065–0.095)	0.083 (0.070–0.097)
Weighted root mean square residual	0.828	0.879

Abbreviation: EB PRO, eating behavior patient‐reported outcome.

#### Reliability

Internal consistency reliability of the EB PRO measure was examined at baseline to assess the consistency of multiple items measuring the same concept. All Cronbach α coefficients were >0.70 (Desire to Eat: 0.852; Capacity to Resist: 0.898; total: 0.926), indicating good‐to‐excellent internal consistency (Table S6). Test–retest reliability of the EB PRO was examined during the screening period at any point (weeks −12 to −1), within the 4 weeks before baseline (weeks −4 to −1), and within the 2 weeks before baseline (weeks −2 to −1). All intraclass correlation coefficients were similar regardless of time period, with values all exceeding 0.70 (Desire to Eat: 0.77, 0.78, and 0.76, respectively; Capacity to Resist: 0.74, 0.74, and 0.77; total: 0.81, 0.81, and 0.80), indicating acceptable test–retest reliability (Table S7).

#### Validity

In order to assess cross‐sectional construct validity (convergent validity), the relationships between the EB PRO measure and selected items and/or domains of PGIS, PGIC, SF‐36v2 PF‐10, TFEQ‐R18V2, and 3‐day food diary were examined. The EB PRO generally showed moderate‐to‐large correlation (Spearman rank correlation coefficient of 0.3–0.7) with PGIS and TFEQ‐R18V2 items and weaker correlation with SF‐36v2 PF‐10 (Table 3), as hypothesized. Correlations with baseline mean daily energy intake (which is shown in Table S8 together with macronutrient intake) were generally weaker, which may have been influenced by the fact that, unlike the EB PRO (7‐day recall period), there was no requirement for the baseline food diary to fall within the 7 days prior to the randomization visit, as well as the high interindividual variability of energy intake. For known‐groups validity, the EB PRO Desire to Eat, Capacity to Resist, and Total Eating Behavior scores were significantly worse with greater severity of hunger measured by PGIS at baseline (Figure 4A), with similar results at weeks 20 and 46 (Table S9). The EB PRO scores were also significantly different according to BMI category at week 46 (Figure 4B), although significance at baseline was nominally missed (Table S10), which should be interpreted cautiously given the potential time difference between the EB PRO recall period and BMI measurement. Similarly, EB PRO scores were significantly different according to PGIC at week 46 (Figure 4C, Table S11).

**TABLE 3:** Convergent validity of the EB PRO measure in the psychometric evaluation study sample.

	EB PRO measure		
	Desire to Eat	Capacity to Resist	Total score
PGIS (baseline)			
Desire to eat	0.61*	0.59*	0.64*
Food cravings	0.65*	0.63*	0.67*
Resisting food cravings	−0.26*	−0.32*	−0.30*
Severity of hunger	0.57*	0.58*	0.61*
Having healthy eating behavior	0.50*	0.54*	0.55*
Overall eating behavior	0.44*	0.49*	0.49*
PGIC (week 46)	0.35*	0.37*	0.37*
SF‐36v2 (baseline)			
PF‐10	−0.23*	−0.27*	−0.26*
TFEQ‐R18V2 (baseline)			
Cognitive restraint/disinhibition	0.20*	0.23*	0.23*
Emotional eating	−0.62*	−0.53*	−0.60*
Item 9 (uncontrolled eating when hungry)	−0.49*	−0.53*	−0.54*
Item 18 (frequency of hunger)	0.43*	0.39*	0.43*
3‐d food diary (baseline)			
Fat	0.12**	0.11**	0.12**
Protein	−0.07	−0.06	−0.07
Carbohydrates	0.06	0.02	0.04
Daily energy intake	0.17**	0.23*	0.21*

*Note*: Data are Spearman rank correlation coefficients.

Abbreviations: EB PRO, eating behavior patient‐reported outcome; PF‐10, 10‐item Physical Functioning scale, acute version, of SF‐36v2; PGIC, Patient Global Impression of Change instrument; PGIS, Patient Global Impression of Severity for Eating Behavior instrument; SF‐36v2, 36‐Item Short‐Form Health Survey version 2; TFEQ‐R18V2, revised 18‐item version of the Three‐Factor Eating Questionnaire.

* *p* < 0.0001.

** *p* < 0.05.

#### Sensitivity to change

**FIGURE 3:**
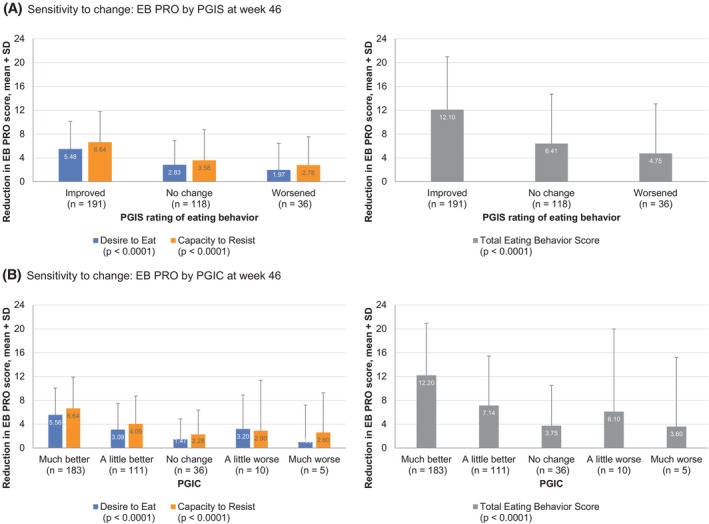
Sensitivity to change of the EB PRO by (A) PGIS and (B) PGIC anchors in the psychometric evaluation study sample. EB PRO, eating behavior patient‐reported outcome; PGIC, Patient Global Impression of Change; PGIS, Patient Global Impression of Severity.

Changes from baseline to week 46 for all EB PRO scores were aligned with changes in PGIS and PGIC (Figure 3). Although each PGIS group (improved, no change, or worsened) had improvements in EB PRO scores at week 46, there was larger improvement in those with improved PGIS (*p* < 0.0001). Similarly, there were larger EB PRO improvements in those with improvements on PGIC (much better, *p* < 0.0001; a little better, *p* < 0.0001). Effect sizes for the EB PRO Total Eating Behavior score were large (PGIS improved: 1.24; PGIC much better: 1.35).

**FIGURE 4:**
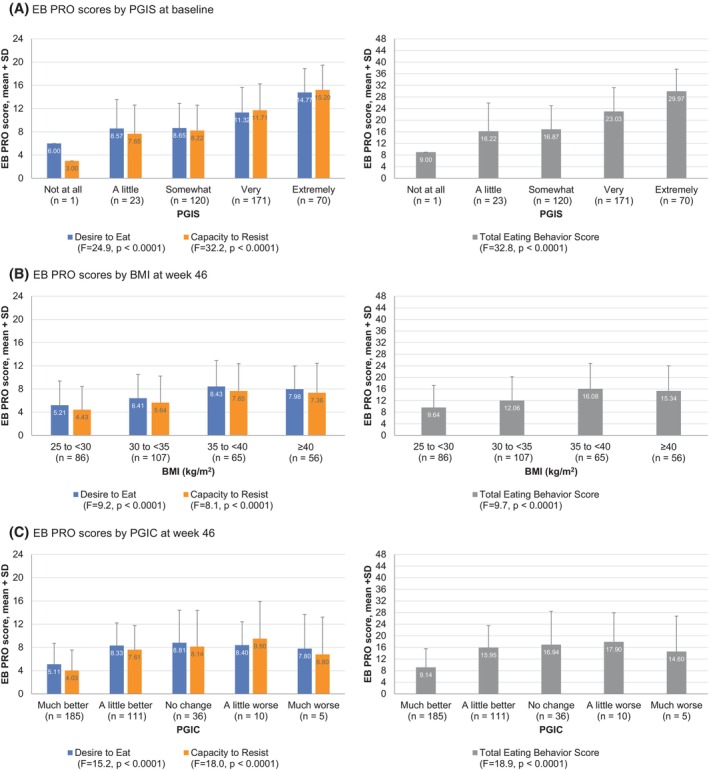
Known‐groups validity in the psychometric evaluation study sample: EB PRO scores by (A) PGIS score, (B) BMI, and (C) PGIC score. EB PRO, eating behavior patient‐reported outcome; PGIC, Patient Global Impression of Change; PGIS, Patient Global Impression of Severity.

#### Interpretation of meaningful change

Anchor‐based assessments of clinically meaningful changes in EB PRO scores generally showed at least moderate correlations with changes in PGIS and PGIC (Table S12). A one‐level improvement in PGIS overall eating behavior score corresponded to reduction (improvement) of 9.2, 4.3, and 4.8 in EB PRO Total Eating Behavior, Desire to Eat, and Capacity to Resist scores, respectively, from baseline to week 46, whereas PGIC of “a little better” corresponded to improvement of 7.1, 3.1, and 4.1, respectively (Figure S1). Furthermore, weight loss of >5% was associated with improvement in Total Eating Behavior score of >8 points at week 46 (Table 4). In the distribution‐based analyses, the one‐half SD at baseline and SE at week 46 were 4.70 and 7.074, respectively, for Total Eating Behavior score, 2.4 and 3.5 for the Desire to Eat domain, and 2.6 and 2.6 for the Capacity to Resist domain.

**TABLE 4:** Mean change in total EB PRO score from baseline to week 46 by weight change in the psychometric evaluation study sample.

Anchors	Total EB score					
	*n*	Adjusted mean change score, mean (SE) (week 46–baseline)	95% CI	*F* value (*p* value)	Effect size (change/SD baseline)	Standardized response (mean change/SD change)
Weight change (baseline to week 46)				7.42 (<0.0001)		
Improve by ≥15%	98	−13.0 (0.89)	−14.78 to −11.30		1.56	1.67
Improve by 10% (10.0%–14.9%)	55	−9.18 (1.18)	−11.51 to −6.86		0.97	1.04
Improve by 5% (5.0%–9.9%)	66	−8.88 (1.08)	−11.00 to −6.76		0.89	0.85
No change (−4.9%–4.9%)	119	−7.31 (0.80)	−8.89 to −5.73		0.77	0.87
Worsen by 5% (5.0%–9.9%)	5	0.00 (3.92)	−7.71 to 7.71		0.00	0.00
Worsen by 10% (10.0%–14.9%)	0					
Worsen by ≥15%	0					
Weight change (baseline to week 46)				8.86 (0.0002)		
Improvement	219	−10.8 (0.60)	−12.00 to −9.64		1.19	1.19
No change	119	−7.31 (0.81)	−8.91 to −5.71		0.77	0.87
Worsening	5	0.00 (3.97)	−7.82 to 7.82		0.00	0.00

*Note:* ANCOVA model run separately for each weight change group.

Abbreviation: EB PRO, eating behavior patient‐reported outcome.

Based on the anchor‐based and distribution‐based findings, clinically meaningful change thresholds for the EB PRO measure were defined as ≥8 points for the Total Eating Behavior score and ≥4 points for each domain.

## DISCUSSION

We have developed and validated a measure (EB PRO) to conveniently and comprehensively evaluate eating behaviors in people living with obesity or overweight. The EB PRO measure comprises 12 items across two domains (“Desire to Eat”: 6 items; “Capacity to Resist”: 6 items) and a Total Eating Behavior score ranging from 0 to 48, with higher scores worse. It typically takes less than 5 min to complete.

The impetus for this work was the substantial burden of obesity (~988 million people globally) [10], its association with altered eating behaviors [1], and the lack of measures to evaluate eating behaviors in the modern obesogenic environment. Historically, eating behavior measures were developed based predominantly on three theories of overeating [15]: the psychosomatic theory of excess eating in response to negative emotional cues such as anxiety [37], the externality theory of hedonic eating in response to external cues regardless of hunger or satiety [38], and the restraint theory that chronic dieting eventually leads to overeating [39]. It is now recognized that eating behaviors can also be a consequence of the disease of obesity rather than a cause [40].

Instruments for measuring eating behavior include the TFEQ [11, 12], the Dutch Eating Behavior Questionnaire [13], the Control of Eating Questionnaire (CoEQ) [22], and several others [15, 16, 17, 18, 19, 20, 21, 22, 23, 24]. Many of these instruments were originally developed for use in people with eating disorders (e.g., TFEQ), focus on only one aspect of eating behaviors such as cravings (CoEQ), and/or are lengthy. Furthermore, many were developed before 2009, when the FDA finalized its guidance on using patient‐reported outcome instruments in clinical trials to support medical product labeling [26, 27, 28], and thereby may not fully align with that guidance. Moreover, verbal reports from people living with obesity are often inconsistent with direct measures of behavior [8, 41, 42], indicating the need for a standardized measure of behavioral attributes rather than relying on anecdotal self‐report.

The current work was conducted in alignment with the 2009 FDA guidance. Concept elicitation involving focus groups of US adults living with obesity or overweight revealed a rich set of eating behavior‐related concepts not comprehensively measured in any existing instrument at that time. This was used to construct a draft conceptual framework and measure comprising domains covering drivers to eat, capacity to resist, and eating habits. Cognitive‐debriefing interviews with a separate set of people living with obesity indicated that the draft measure evaluates eating behaviors and associated domains, and that the items and their domains are relevant and important to people living with obesity, appropriate and comprehensive, and clear and understandable.

In psychometric evaluation using a sample of people living with obesity who participated in a recent clinical trial of an investigational AOM, the draft conceptual framework and measure were refined to six items each within the domains of Desire to Eat and Capacity to Resist, based on item correlations and factor analyses. The domain of Eating Habits/Homeostatic was removed owing to poor fit with the overall model (low reliability and correlation with the other items). The EB PRO measure displayed good internal consistency and test–test reliability. As expected, correlation was generally moderate to large with PGIS and TFEQ‐R18V2 and weaker with SF‐36v2 PF10, whereas scores significantly differed based on PGIS‐assessed hunger and body weight. The measure's sensitivity to change was demonstrated by alignment to changes in PGIS and PGIC scores and weight loss. The items in the final EB PRO measure encompass concepts such as eating when emotional, when bored, for comfort, and during social interactions, as well as in response to food cues and hedonic eating. The Capacity to Resist domain may encompass concepts similar to self‐efficacy, i.e., an individual's belief in his or her ability to self‐regulate eating [43]. As the confidence to control eating in obesogenic environments, eating self‐efficacy may play an important role in weight loss and weight maintenance if it translates to favorable eating behaviors. A detailed comparison of the potential relationship between the Capacity to Resist domain of the EB PRO measure and eating self‐efficacy may be a productive focus for future research.

Limitations of this work include that the qualitative concept elicitation and cognitive debriefing were conducted exclusively in the United States; however, the psychometric validation was conducted using data from a multinational clinical trial, suggesting that the measure is generalizable to people living with obesity in other countries. Although men and women were generally represented equally in the concept‐elicitation and cognitive‐debriefing qualitative studies, there was a slight underrepresentation of men in the psychometric evaluation study (32%). Furthermore, as the studies were all conducted in adults, the EB PRO measure has not been validated for use in children or adolescents.

## CONCLUSION

The rapidly rising rate of both obesity and effective new pharmacological treatments for this disease means that assessment of eating behaviors is increasingly important. The EB PRO measure developed here has psychometric properties suggesting a promising tool for comprehensively assessing eating behaviors in people living with obesity or overweight in the modern obesogenic environment. It can be used in both routine care and clinical trials and may support medication labeling according to US FDA guidance.

## CONFLICT OF INTEREST STATEMENT

Donald M. Bushnell and Meryl Brod are paid consultants to the pharmaceutical industry, including Boehringer Ingelheim. Shirley Fung was a paid consultant to the pharmaceutical industry when working on the project; Shirley Fung is currently working at the Centers for Medicare and Medicaid Services and declares no conflicts of interest. Carl A. Roberts has received grant funding from Unilever that is unrelated to the current work and acted as paid consultant for Boehringer Ingelheim. Carel W. Le Roux has received personal fees from Boehringer Ingelheim, Morphic Medical (formerly GI Dynamics), Herbalife Nutrition Ltd., Johnson & Johnson, Keyron, Eli Lilly and Company, and Novo Nordisk A/S outside the submitted work. Oren Steen has received research support from Alnylam Pharmaceuticals, Inc., Anji Pharma, AstraZeneca plc, Boehringer Ingelheim, CRISPR Therapeutics AG, Eli Lilly and Company, Gilead Sciences, Inc., Janssen Pharmaceuticals, Kowa Company, Ltd., Medicago, Moderna, Inc., Novavax, Inc., Novartis, Novo Nordisk A/S, Pfizer Inc., Sanofi SA, ViaCyte, and Zucara Therapeutics Inc.; speakers' bureau fees from Abbott Laboratories, Amgen Inc., AstraZeneca plc, Bausch Health Companies Inc., Boehringer Ingelheim, Eli Lilly and Company, HLS Therapeutics, Janssen Pharmaceuticals, LMC Healthcare, Novo Nordisk A/S, Pfizer Inc., and Sanofi SA; and consultancy fees from Amgen Inc., Bayer AG, Eli Lilly and Company, HLS, Novo Nordisk A/S, and Sanofi SA. Anita M. Hennige and Anastasia Uster are employees of Boehringer Ingelheim. Kathryn J. Lucas declared no conflicts of interest.

## Supporting information


Data S1.

## Data Availability

The eating behavior patient‐reported outcome (EB PRO) measure can be made available for research purposes upon request.
