# Supplementary material for: Development and validation of an eating behavior patient‐reported outcome measure in people living with obesity or overweight
Source: Obesity (Silver Spring). 2025 May 28;33(7):1249–62. doi: 10.1002/oby.24299 (PMC12210104; doi:10.1002/oby.24299)
Supplement: Supplementary file 1 — Data S1. [file OBY-33-1249-s001.docx]

**Supporting information**

**Contents**

[**Table S1** Demographic and clinical characteristics of concept-elicitation participants 2](#_Toc190784337)

[**Table S2** Demographic and clinical characteristics of cognitive-debriefing participants 4](#_Toc190784338)

[**Table S3** Item response distributions for the draft EB PRO measure in psychometric analyses. 6](#_Toc190784339)

[**Table S4** Item score by time point for the draft EB PRO measure in psychometric analyses. 10](#_Toc190784340)

[**Table S5** Item-item and item-total correlation coefficients for the draft EB PRO measure 11](#_Toc190784341)

[**Table S6** Internal consistency reliability of the EB PRO measure at baseline 13](#_Toc190784342)

[**Table S7** Test-test reliability of the EB PRO measure during screening^a^ 14](#_Toc190784343)

[**Table S8** Baseline daily energy and macronutrient intake based on three-day food diaries 16](#_Toc190784344)

[**Table S9** Known-groups validity: EB PRO score by PGIS 17](#_Toc190784345)

[**Table S10** Known-groups validity: EB PRO score by BMI 18](#_Toc190784346)

[**Table S11** Known-groups validity: EB PRO score by PGIC 19](#_Toc190784347)

[**Table S12** Change-score correlations of the EB PRO and PGIS and PGIC 20](#_Toc190784348)

[**Figure S1** Anchor-based analysis of meaningful change threshold by (A) PGIS score and (B) PGIC score. 21](#_Toc190784349)

#### **Table S1** Demographic and clinical characteristics of concept-elicitation participants

| **Characteristic** | **BMI (kg/m^2^)** | | |  |
| --- | --- | --- | --- | --- |
|  | **27 to <30**  **(*n* = 13)** | **30 to <40**  **(*n* = 19)** | **≥40**  **(*n* = 21)** | **Total**  **(*N* = 53)** |
| Age (years), mean (SD) | 53.2 (12.08) | 51.6 (14.45) | 47.9 (12.81) | 50.5 (13.19) |
| Gender, *n* (%) |  |  |  |  |
| Male | 6 (46.2) | 11 (57.9) | 10 (47.6) | 27 (50.9) |
| Female | 7 (53.8) | 8 (42.1) | 11 (52.4) | 26 (49.1) |
| Race, *n* (%) |  |  |  |  |
| White | 9 (69.2) | 9 (47.4) | 7 (33.3) | 25 (47.2) |
| Black/African American | 3 (23.1) | 9 (47.4) | 14 (66.7) | 26 (49.1) |
| Asian | 0 | 0 | 0 | 0 |
| Native Hawaiian/Pacific Islander | 0 | 0 | 0 | 0 |
| American Indian/Alaska Native | 0 | 0 | 0 | 0 |
| Other | 1 (7.7) | 1 (5.3) | 0 | 2 (3.8) |
| Ethnicity, *n* (%) |  |  |  |  |
| Hispanic/Latino | 2 (15.4) | 3 (15.8) | 2 (9.5) | 7 (13.2) |
| Not Hispanic/Latino | 11 (84.6) | 16 (84.2) | 19 (90.5) | 46 (86.8) |
| Living status, *n* (%) |  |  |  |  |
| Alone | 3 (23.1) | 4 (21.1) | 6 (28.6) | 13 (24.5) |
| With partner/spouse, family, or friends | 10 (76.9) | 14 (73.7) | 13 (61.9) | 37 (69.8) |
| Other | 0 | 1 (5.3) | 2 (9.5) | 3 (5.7) |
| Employment status, *n* (%) |  |  |  |  |
| Employed, full-time | 7 (53.8) | 12 (63.2) | 13 (61.9) | 32 (60.4) |
| Employed, part-time | 3 (23.1) | 1 (5.3) | 1 (4.8) | 5 (9.4) |
| Homemaker | 1 (7.7) | 0 | 1 (4.8) | 2 (3.8) |
| Student | 0 | 0 | 0 | 0 |
| Unemployed | 1 (7.7) | 1 (5.3) | 1 (4.8) | 3 (5.7) |
| Retired | 1 (7.7) | 4 (21.1) | 4 (19.0) | 9 (17.0) |
| Disabled | 0 | 1 (5.3) | 4 (19.0) | 5 (9.4) |
| Highest level of education, *n* (%) |  |  |  |  |
| Elementary/primary school | 0 | 0 | 0 | 0 |
| Secondary/high school | 2 (15.4) | 2 (10.5) | 4 (19.0) | 8 (15.1) |
| Associate/technical/vocational degree | 1 (7.7) | 0 | 2 (9.5) | 3 (5.7) |
| Some college | 4 (30.8) | 5 (26.3) | 7 (33.3) | 16 (30.2) |
| College degree | 3 (23.1) | 9 (47.4) | 5 (23.8) | 17 (32.1) |
| Graduate degree | 3 (23.1) | 4 (21.1) | 5 (23.8) | 12 (22.6) |
| Other | 0 | 0 | 1 (4.8) | 1 (1.9) |
| Overall health, *n* (%) |  |  |  |  |
| Excellent | 2 (15.4) | 1 (5.3) | 0 | 3 (5.7) |
| Fair | 1 (7.7) | 5 (26.3) | 2 (9.5) | 8 (15.1) |
| Average | 9 (69.2) | 9 (47.4) | 10 (47.6) | 28 (52.8) |
| Poor | 1 (7.7) | 4 (21.1) | 8 (38.1) | 13 (24.5) |
| Very poor | 0 | 0 | 1 (4.8) | 1 (1.9) |
| BMI (kg/m^2^), mean (SD) | 28.4 (1.04) | 35.0 (2.94) | 48.1 (4.50) | 38.6 (8.85) |
| Existing health conditions, *n* (%) |  |  |  |  |
| Type 2 diabetes | 4 (30.8) | 6 (31.6) | 8 (38.1) | 18 (34.0) |
| Heart disease | 0 | 2 (10.5) | 2 (9.5) | 4 (7.5) |
| Stroke | 0 | 0 | 2 (9.5) | 2 (3.8) |
| High blood pressure | 5 (38.5) | 12 (63.2) | 13 (61.9) | 30 (56.6) |
| High cholesterol | 8 (61.5) | 5 (26.3) | 9 (42.9) | 22 (41.5) |
| Sleep apnea | 1 (7.7) | 3 (15.8) | 9 (42.9) | 13 (24.5) |
| Urinary incontinence | 0 | 1 (5.3) | 4 (19.0) | 5 (9.4) |
| Pulmonary dysfunction | 0 | 0 | 0 | 0 |
| Osteoarthritis | 0 | 1 (5.3) | 6 (28.6) | 7 (13.2) |
| Back and neck pain | 4 (30.8) | 8 (42.1) | 12 (57.1) | 24 (45.3) |
| Other pain | 2 (15.4) | 3 (15.8) | 7 (33.3) | 12 (22.6) |
| Other | 3 (23.1) | 3 (15.8) | 3 (14.3) | 9 (17.0) |
| No other health conditions | 0 | 0 | 1 (4.8) | 1 (1.9) |
| *Note*: Data shown as mean (SD) for continuous variables and *n* (%) for categorical variables.  Abbreviations: BMI, body mass index; SD, standard deviation. | | | | |

#### **Table S2** Demographic and clinical characteristics of cognitive-debriefing participants

| **Characteristic** | **BMI (kg/m^2^)** | | |  |
| --- | --- | --- | --- | --- |
|  | **27 to <30**  **(*n* = 2)** | **30 to <40**  **(*n* = 7)** | **≥40**  **(*n* = 6)** | **Total**  **(*N* = 15)** |
| Age (years), mean (SD) | 64.5 (3.5) | 48.0 (13.7) | 46.7 (6.3) | 49.7 (11.5) |
| Gender, *n* (%) |  |  |  |  |
| Male | 1 (50.0) | 3 (42.9) | 3 (50.0) | 7 (46.7) |
| Female | 1 (50.0) | 4 (57.1) | 3 (50.0) | 8 (53.3) |
| Race, *n* (%) |  |  |  |  |
| White | 2 (100) | 6 (85.7) | 5 (83.3) | 13 (86.7) |
| Black/African American | 0 | 1 (14.3) | 1 (16.7) | 2 (13.3) |
| Ethnicity, *n* (%) |  |  |  |  |
| Not Hispanic/Latino | 2 (100) | 7 (100) | 6 (100) | 15 (100) |
| Living status, *n* (%) |  |  |  |  |
| Alone | 0 | 1 (14.3) | 1 (16.7) | 2 (13.3) |
| With partner/spouse, family, or friends | 2 (100) | 6 (85.7) | 5 (83.3) | 13 (86.7) |
| Employment status, *n* (%) |  |  |  |  |
| Employed, full-time | 1 (50.0) | 4 (57.1) | 3 (50.0) | 8 (53.3) |
| Employed, part-time | 0 | 1 (14.3) | 2 (33.3) | 3 (20.0) |
| Homemaker | 0 | 1 (14.3) | 0 | 1 (6.7) |
| Student | 0 | 0 | 1 (16.7) | 1 (6.7) |
| Unemployed | 1 (50.0) | 0 | 0 | 1 (6.7) |
| Retired | 0 | 1 (14.3) | 0 | 1 (6.7) |
| Highest level of education, *n* (%) |  |  |  |  |
| Some college | 0 | 0 | 2 (33.3) | 2 (13.3) |
| College degree | 0 | 4 (57.1) | 2 (33.3) | 6 (40.0) |
| Graduate degree | 2 (100) | 3 (42.9) | 2 (33.3) | 7 (46.7) |
| Overall health, *n* (%) |  |  |  |  |
| Fair | 1 (50.0) | 2 (28.6) | 1 (16.7) | 4 (26.7) |
| Average | 1 (50.0) | 2 (28.6) | 1 (16.7) | 4 (26.7) |
| Poor | 0 | 3 (42.9) | 4 (66.7) | 7 (46.7) |
| BMI (kg/m^2^), mean (SD) | 28.8 (0.2) | 32.8 (2.6) | 43.0 (2.1) | 36.3 (6.1) |
| Existing health conditions, *n* (%) |  |  |  |  |
| Type 2 diabetes | 1 (50.0) | 1 (14.3) | 1 (16.7) | 3 (20.0) |
| Heart disease | 0 | 1 (14.3) | 0 | 1 (6.7) |
| Stroke | 0 | 1 (14.3) | 0 | 1 (6.7) |
| High blood pressure | 2 (100) | 2 (28.6) | 3 (50.0) | 7 (46.7) |
| High cholesterol | 2 (100) | 3 (42.9) | 1 (16.7) | 6 (40.0) |
| Sleep apnea | 0 | 2 (28.6) | 3 (50.0) | 5 (33.3) |
| Urinary incontinence | 0 | 0 | 3 (50.0) | 3 (20.0) |
| Osteoarthritis | 1 (50.0) | 1 (14.3) | 1 (16.7) | 3 (20.0) |
| Back and neck pain | 1 (50.0) | 2 (28.6) | 2 (33.3) | 5 (33.3) |
| Other pain | 1 (50.0) | 2 (28.6) | 1 (16.7) | 4 (26.7) |
| Other | 1 (50.0) | 0 | 0 | 1 (6.7) |
| No other health conditions | 0 | 2 (28.6) | 1 (16.7) | 3 (20.0) |
| *Note*: Data shown as mean (SD) for continuous variables and *n* (%) for categorical variables.  Abbreviations: BMI, body mass index; SD, standard deviation. | | | | |

#### **Table S3** Item response distributions for the draft EB PRO measure in psychometric analyses.

| ***n* (%)** | **Screening**  **(*N* = 386)** | **Week 0**  **(*N* = 385)** | **Week 20**  **(*N* = 288)** | **Week 46**  **(*N* = 348)** |
| --- | --- | --- | --- | --- |
| Item 1: Feeling emotional |  |  |  |  |
| 0: Not at all | 81 (21.0) | 73 (19.0) | 119 (41.3) | 133 (38.2) |
| 1: A little | 86 (22.3) | 95 (24.7) | 103 (35.8) | 120 (34.5) |
| 2: Moderately | 106 (27.5) | 120 (31.2) | 45 (15.6) | 68 (19.5) |
| 3: A lot | 94 (24.4) | 84 (21.8) | 19 (6.6) | 25 (7.2) |
| 4: Extremely | 19 (4.9) | 13 (3.4) | 2 (0.7) | 2 (0.6) |
| Item 2: Eating with family and friends |  |  |  |  |
| 0: Not at all | 28 (7.3) | 23 (6.0) | 45 (15.6) | 64 (18.4) |
| 1: A little | 61 (15.8) | 58 (15.1) | 110 (38.2) | 102 (29.3) |
| 2: Moderately | 177 (46.0) | 174 (45.2) | 105 (36.5) | 139 (39.9) |
| 3: A lot | 109 (28.3) | 115 (29.9) | 27 (9.4) | 40 (11.5) |
| 4: Extremely | 10 (2.6) | 15 (3.9) | 1 (0.3) | 3 (0.9) |
| Item 3: Eating socially (removed from final measure) |  |  |  |  |
| 0: Not at all | 72 (18.8) | 88 (22.9) | 91 (31.8) | 89 (25.6) |
| 1: A little | 68 (17.8) | 68 (17.7) | 104 (36.4) | 113 (32.5) |
| 2: Moderately | 159 (41.5) | 143 (37.2) | 72 (25.2) | 111 (31.9) |
| 3: A lot | 71 (18.5) | 76 (19.8) | 17 (5.9) | 31 (8.9) |
| 4: Extremely | 13 (3.4) | 9 (2.3) | 2 (0.7) | 4 (1.1) |
| Item 4: When hungry (removed from final measure) |  |  |  |  |
| 0: Not at all | 6 (1.6) | 6 (1.6) | 24 (8.3) | 14 (4.0) |
| 1: A little | 22 (5.7) | 44 (11.4) | 90 (31.3) | 96 (27.6) |
| 2: Moderately | 134 (34.9) | 127 (33.0) | 122 (42.4) | 162 (46.6) |
| 3: A lot | 189 (49.2) | 177 (46.0) | 44 (15.3) | 67 (19.3) |
| 4: Extremely | 33 (8.6) | 31 (8.1) | 8 (2.8) | 9 (2.6) |
| Item 5: Indulge in comfort foods |  |  |  |  |
| 0: Not at all | 45 (11.7) | 43 (11.2) | 81 (28.1) | 95 (27.3) |
| 1: A little | 91 (23.7) | 98 (25.5) | 132 (45.8) | 142 (40.8) |
| 2: Moderately | 115 (29.9) | 131 (34.0) | 52 (18.1) | 79 (22.7) |
| 3: A lot | 105 (27.3) | 89 (23.1) | 21 (7.3) | 30 (8.6) |
| 4: Extremely | 28 (7.3) | 24 (6.2) | 2 (0.7) | 2 (0.6) |
| Item 6: When you were bored |  |  |  |  |
| 0: Not at all | 70 (18.2) | 74 (19.2) | 141 (49.0) | 148 (42.5) |
| 1: A little | 99 (25.8) | 109 (28.3) | 94 (32.6) | 122 (35.1) |
| 2: Moderately | 101 (26.3) | 108 (28.1) | 35 (12.2) | 54 (15.5) |
| 3: A lot | 94 (24.5) | 81 (21.0) | 15 (5.2) | 23 (6.6) |
| 4: Extremely | 20 (5.2) | 13 (3.4) | 3 (1.0) | 1 (0.3) |
| Item 7: Eat your favorite unhealthy foods |  |  |  |  |
| 0: Not at all | 27 (7.0) | 22 (5.7) | 64 (22.2) | 75 (21.6) |
| 1: A little | 88 (22.9) | 111 (28.8) | 147 (51.0) | 158 (45.4) |
| 2: Moderately | 117 (30.5) | 127 (33.0) | 60 (20.8) | 89 (25.6) |
| 3: A lot | 125 (32.6) | 105 (27.3) | 12 (4.2) | 24 (6.9) |
| 4: Extremely | 27 (7.0) | 20 (5.2) | 5 (1.7) | 2 (0.6) |
| Item 8: Eat foods readily available |  |  |  |  |
| 0: Not at all | 67 (17.4) | 66 (17.1) | 109 (37.8) | 119 (34.3) |
| 1: A little | 90 (23.4) | 91 (23.6) | 103 (35.8) | 127 (36.6) |
| 2: Moderately | 131 (34.1) | 130 (33.8) | 59 (20.5) | 73 (21.0) |
| 3: A lot | 82 (21.4) | 86 (22.3) | 14 (4.9) | 26 (7.5) |
| 4: Extremely | 14 (3.6) | 12 (3.1) | 3 (1.0) | 2 (0.6) |
| Item 9: Foods addictive (removed from final measure) |  |  |  |  |
| 0: Not at all | 105 (27.3) | 120 (31.2) | 155 (53.8) | 184 (53.0) |
| 1: A little | 88 (22.9) | 76 (19.7) | 89 (30.9) | 87 (25.1) |
| 2: Moderately | 96 (25.0) | 91 (23.6) | 30 (10.4) | 48 (13.8) |
| 3: A lot | 76 (19.8) | 80 (20.8) | 9 (3.1) | 22 (6.3) |
| 4: Extremely | 19 (4.9) | 18 (4.7) | 5 (1.7) | 6 (1.7) |
| Item 10: Controlled by food (removed from final measure) |  |  |  |  |
| 0: Not at all | 146 (38.0) | 149 (38.7) | 195 (67.7) | 215 (62.0) |
| 1: A little | 85 (22.1) | 91 (23.6) | 59 (20.5) | 81 (23.3) |
| 2: Moderately | 80 (20.8) | 80 (20.8) | 20 (6.9) | 32 (9.2) |
| 3: A lot | 58 (15.1) | 59 (15.3) | 10 (3.5) | 16 (4.6) |
| 4: Extremely | 15 (3.9) | 6 (1.6) | 4 (1.4) | 3 (0.9) |
| Item 11: Fight against eating habits |  |  |  |  |
| 0: Not at all | 43 (11.2) | 52 (13.5) | 96 (33.3) | 110 (31.7) |
| 1: A little | 95 (24.7) | 87 (22.6) | 128 (44.4) | 143 (41.2) |
| 2: Moderately | 118 (30.7) | 109 (28.3) | 43 (14.9) | 57 (16.4) |
| 3: A lot | 95 (24.7) | 118 (30.6) | 17 (5.9) | 31 (8.9) |
| 4: Extremely | 33 (8.6) | 19 (4.9) | 4 (1.4) | 6 (1.7) |
| Item 12: Make good food choices |  |  |  |  |
| 0: Not at all | 35 (9.1) | 32 (8.3) | 87 (30.2) | 103 (29.6) |
| 1: A little | 100 (26.0) | 99 (25.7) | 121 (42.0) | 140 (40.2) |
| 2: Moderately | 159 (41.3) | 155 (40.3) | 59 (20.5) | 82 (23.6) |
| 3: A lot | 78 (20.3) | 89 (23.1) | 19 (6.6) | 20 (5.7) |
| 4: Extremely | 13 (3.4) | 10 (2.6) | 2 (0.7) | 3 (0.9) |
| Item 13: Control portion size |  |  |  |  |
| 0: Not at all | 40 (10.4) | 48 (12.5) | 147 (51.0) | 164 (47.1) |
| 1: A little | 84 (21.8) | 90 (23.4) | 86 (29.9) | 115 (33.0) |
| 2: Moderately | 143 (37.1) | 133 (34.5) | 42 (14.6) | 44 (12.6) |
| 3: A lot | 91 (23.6) | 100 (26.0) | 10 (3.5) | 23 (6.6) |
| 4: Extremely | 27 (7.0) | 14 (3.6) | 3 (1.0) | 2 (0.6) |
| Item 14: Control eating when hungry |  |  |  |  |
| 0: Not at all | 36 (9.4) | 36 (9.4) | 104 (36.1) | 108 (31.0) |
| 1: A little | 73 (19.0) | 100 (26.0) | 114 (39.6) | 140 (40.2) |
| 2: Moderately | 141 (36.6) | 127 (33.0) | 50 (17.4) | 70 (20.1) |
| 3: A lot | 114 (29.6) | 102 (26.5) | 18 (6.3) | 29 (8.3) |
| 4: Extremely | 21 (5.5) | 20 (5.2) | 2 (0.7) | 1 (0.3) |
| Item 15: Avoid buying foods resist |  |  |  |  |
| 0: Not at all | 66 (17.1) | 71 (18.4) | 144 (50.0) | 158 (45.4) |
| 1: A little | 106 (27.5) | 111 (28.8) | 101 (35.1) | 118 (33.9) |
| 2: Moderately | 104 (27.0) | 114 (29.6) | 30 (10.4) | 55 (15.8) |
| 3: A lot | 90 (23.4) | 75 (19.5) | 11 (3.8) | 15 (4.3) |
| 4: Extremely | 19 (4.9) | 14 (3.6) | 2 (0.7) | 2 (0.6) |
| Item 16: Avoid eating foods resist |  |  |  |  |
| 0: Not at all | 36 (9.4) | 39 (10.1) | 94 (32.6) | 114 (32.8) |
| 1: A little | 114 (29.6) | 109 (28.3) | 140 (48.6) | 150 (43.1) |
| 2: Moderately | 120 (31.2) | 123 (31.9) | 43 (14.9) | 58 (16.7) |
| 3: A lot | 92 (23.9) | 102 (26.5) | 9 (3.1) | 25 (7.2) |
| 4: Extremely | 23 (6.0) | 12 (3.1) | 2 (0.7) | 1 (0.3) |
| Item 17: Difficulty resisting (removed from final measure) |  |  |  |  |
| 0: Not at all | 36 (9.4) | 47 (12.2) | 133 (46.2) | 143 (41.1) |
| 1: A little | 111 (28.8) | 111 (28.8) | 105 (36.5) | 123 (35.3) |
| 2: Moderately | 132 (34.3) | 122 (31.7) | 38 (13.2) | 62 (17.8) |
| 3: A lot | 91 (23.6) | 91 (23.6) | 10 (3.5) | 18 (5.2) |
| 4: Extremely | 15 (3.9) | 14 (3.6) | 2 (0.7) | 2 (0.6) |
| Item 18: Regular meals (removed from final measure) |  |  |  |  |
| 0: Never | 24 (6.2) | 22 (5.7) | 17 (5.9) | 13 (3.7) |
| 1: Rarely | 88 (22.9) | 82 (21.3) | 36 (12.5) | 52 (14.9) |
| 2: Sometimes | 92 (23.9) | 102 (26.5) | 48 (16.7) | 83 (23.9) |
| 3: Often | 137 (35.6) | 134 (34.8) | 127 (44.1) | 145 (41.7) |
| 4: Always | 44 (11.4) | 45 (11.7) | 60 (20.8) | 55 (15.8) |
| Item 19: Snacked during day (removed from final measure) |  |  |  |  |
| 0: Never | 19 (4.9) | 16 (4.2) | 29 (10.1) | 35 (10.1) |
| 1: Rarely | 72 (18.7) | 93 (24.2) | 130 (45.1) | 138 (39.7) |
| 2: Sometimes | 148 (38.4) | 157 (40.8) | 91 (31.6) | 113 (32.5) |
| 3: Often | 110 (28.6) | 98 (25.5) | 27 (9.4) | 53 (15.2) |
| 4: Always | 36 (9.4) | 21 (5.5) | 11 (3.8) | 9 (2.6) |
| Item 20: Snacked during evening/night (removed from final measure) |  |  |  |  |
| 0: Never | 49 (12.7) | 61 (15.8) | 87 (30.2) | 120 (34.6) |
| 1: Rarely | 107 (27.8) | 106 (27.5) | 114 (39.6) | 129 (37.2) |
| 2: Sometimes | 116 (30.1) | 108 (28.1) | 65 (22.6) | 64 (18.4) |
| 3: Often | 83 (21.6) | 87 (22.6) | 18 (6.3) | 29 (8.4) |
| 4: Always | 30 (7.8) | 23 (6.0) | 4 (1.4) | 5 (1.4) |
| Item 21: Felt hungry (removed from final measure) |  |  |  |  |
| 0: Never | 6 (1.6) | 6 (1.6) | 27 (9.4) | 18 (5.2) |
| 1: Rarely | 46 (11.9) | 45 (11.7) | 91 (31.6) | 94 (27.1) |
| 2: Sometimes | 188 (48.8) | 200 (51.9) | 141 (49.0) | 188 (54.2) |
| 3: Often | 129 (33.5) | 122 (31.7) | 26 (9.0) | 40 (11.5) |
| 4: Always | 16 (4.2) | 12 (3.1) | 3 (1.0) | 7 (2.0) |
| Item 22: Felt full (removed from final measure) |  |  |  |  |
| 0: Never | 7 (1.8) | 4 (1.0) | 3 (1.0) | 3 (0.9) |
| 1: Rarely | 21 (5.5) | 28 (7.3) | 16 (5.6) | 12 (3.5) |
| 2: Sometimes | 131 (34.0) | 111 (28.8) | 38 (13.2) | 73 (21.0) |
| 3: Often | 165 (42.9) | 179 (46.5) | 131 (45.5) | 160 (46.1) |
| 4: Always | 61 (15.8) | 63 (16.4) | 100 (34.7) | 99 (28.5) |
| Item 23: Satisfied by meal (removed from final measure) |  |  |  |  |
| 0: Never | 5 (1.3) | 4 (1.0) | 7 (2.4) | 6 (1.7) |
| 1: Rarely | 32 (8.3) | 25 (6.5) | 11 (3.8) | 13 (3.7) |
| 2: Sometimes | 100 (26.0) | 118 (30.6) | 36 (12.5) | 46 (13.3) |
| 3: Often | 183 (47.5) | 167 (43.4) | 116 (40.3) | 156 (45.0) |
| 4: Always | 65 (16.9) | 71 (18.4) | 118 (41.0) | 126 (36.3) |
| Abbreviations: EB PRO, Eating Behavior Patient-Reported Outcome. | | | | |

#### **Table S4** Item score by time point for the draft EB PRO measure in psychometric analyses.

| **Item (*n* at screening, baseline, week 20, week 46)** | **Mean (SD)** | | | |
| --- | --- | --- | --- | --- |
|  | **Screening** | **Baseline** | **Week 20** | **Week 46** |
| 1: Feeling emotional (386, 385, 288, 348) | 1.7 (1.19) | 1.7 (1.12) | 0.9 (0.94) | 1.0 (0.96) |
| 2: Eating with family and friends (385, 385, 288, 348) | 2.0 (0.92) | 2.1 (0.91) | 1.4 (0.87) | 1.5 (0.95) |
| 3: Eating socially (383, 384, 286, 348)^a^ | 1.7 (1.08) | 1.6 (1.11) | 1.1 (0.93) | 1.3 (0.98) |
| 4: When hungry (384, 385, 288, 348)^a^ | 2.6 (0.79) | 2.5 (0.86) | 1.7 (0.92) | 1.9 (0.85) |
| 5: Indulge in comfort foods (384, 385, 288, 348) | 1.9 (1.13) | 1.9 (1.08) | 1.1 (0.90) | 1.1 (0.94) |
| 6: When you were bored (384, 385, 288, 348) | 1.7 (1.17) | 1.6 (1.12) | 0.8 (0.93) | 0.9 (0.93) |
| 7: Eat your favorite unhealthy foods (384, 385, 288, 348) | 2.1 (1.05) | 2.0 (1.00) | 1.1 (0.86) | 1.2 (0.87) |
| 8: Eat foods readily available (384, 385, 288, 347) | 1.7 (1.10) | 1.7 (1.09) | 1.0 (0.93) | 1.0 (0.95) |
| 9: Foods addictive (384, 385, 288, 347)^a^ | 1.5 (1.22) | 1.5 (1.25) | 0.7 (0.91) | 0.8 (1.02) |
| 10: Controlled by food (384, 385, 288, 347)^a^ | 1.2 (1.22) | 1.2 (1.15) | 0.5 (0.88) | 0.6 (0.90) |
| 11: Fight against eating habits (384, 385, 288, 347) | 1.9 (1.13) | 1.9 (1.12) | 1.0 (0.92) | 1.1 (1.00) |
| 12: Make good food choices (385, 385, 288, 348) | 1.8 (0.97) | 1.9 (0.95) | 1.1 (0.91) | 1.1 (0.91) |
| 13: Control portion size (385, 385, 288, 348) | 2.0 (1.07) | 1.8 (1.06) | 0.7 (0.91) | 0.8 (0.94) |
| 14: Control eating when hungry (385, 385, 288, 348) | 2.0 (1.04) | 1.9 (1.05) | 1.0 (0.92) | 1.1 (0.93) |
| 15: Avoid buying foods resist (385, 385, 288, 348) | 1.7 (1.15) | 1.6 (1.10) | 0.7 (0.86) | 0.8 (0.90) |
| 16: Avoid eating foods resist (385, 385, 288, 348) | 1.9 (1.07) | 1.8 (1.03) | 0.9 (0.81) | 1.0 (0.90) |
| 17: Difficulty resisting (385, 385, 288, 348)^a^ | 1.8 (1.02) | 1.8 (1.05) | 0.8 (0.86) | 0.9 (0.92) |
| 18: Regular meals (385, 385, 288, 348)^a^ | 1.8 (1.11) | 1.7 (1.09) | 1.4 (1.12) | 1.5 (1.05) |
| 19: Snacked during day (385, 385, 288, 348)^a^ | 2.2 (1.01) | 2.0 (0.94) | 1.5 (0.93) | 1.6 (0.95) |
| 20: Snacked during evening/night (385, 385, 288, 347)^a^ | 1.8 (1.14) | 1.8 (1.15) | 1.1 (0.95) | 1.0 (1.00) |
| 21: Felt hungry (385, 385, 288, 347)^a^ | 2.3 (0.78) | 2.2 (0.75) | 1.6 (0.82) | 1.8 (0.79) |
| 22: Felt full (385, 385, 288, 347)^a^ | 1.3 (0.87) | 1.3 (0.86) | 0.9 (0.89) | 1.0 (0.84) |
| 23: Satisfied by meal (385, 385, 288, 347)^a^ | 1.3 (0.89) | 1.3 (0.88) | 0.9 (0.94) | 0.9 (0.89) |
| Abbreviations: EB PRO, Eating Behavior Patient-Reported Outcome; SD, standard deviation.  ^a^Removed from final measure. | | | | |

#### **Table S5** Item-item and item-total correlation coefficients for the draft EB PRO measure

**A Screening**


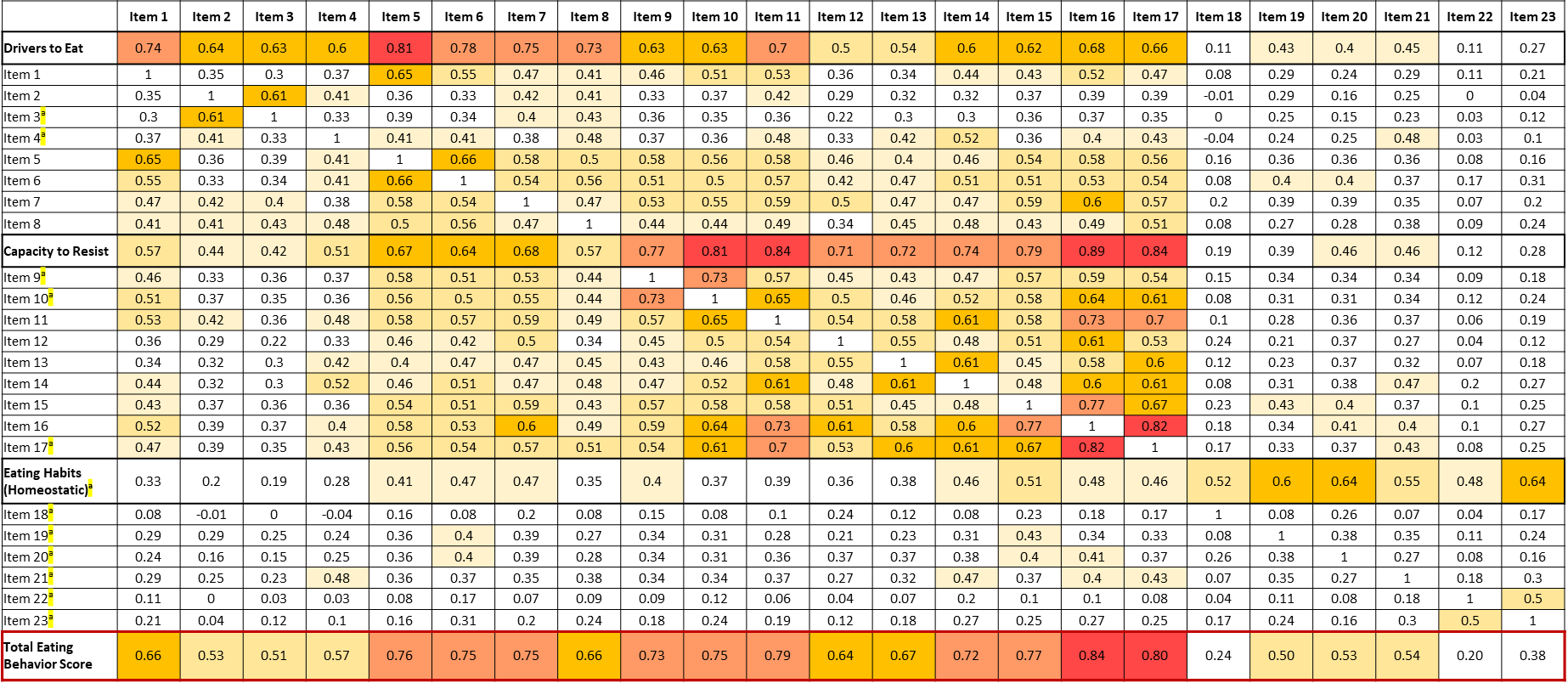


**B Baseline**


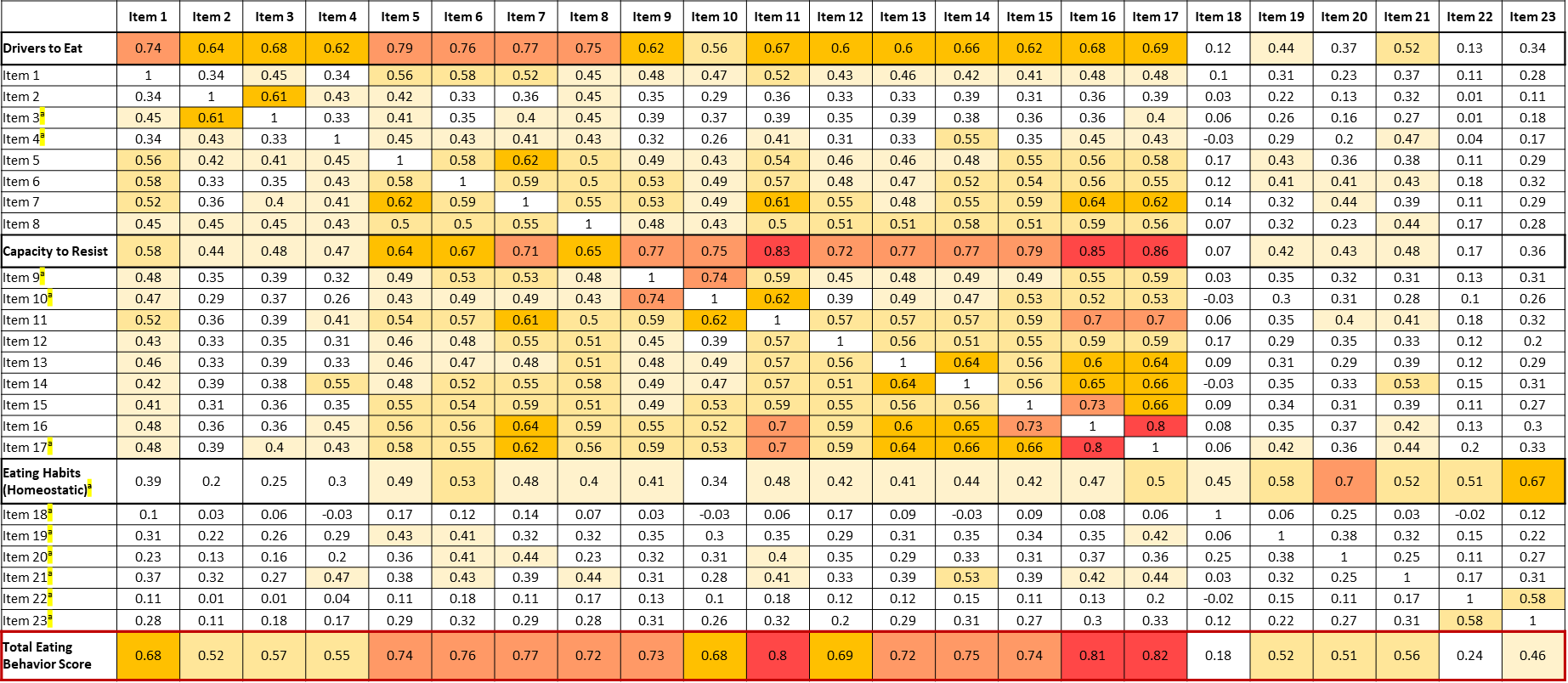


*Note*: highlighted cells show the strength of correlations: moderate (light yellow) to strong (red).

Abbreviation: EB PRO, Eating Behavior Patient-Reported Outcome.

^a^Removed from final measure.

#### **Table S6** Internal consistency reliability of the EB PRO measure at baseline

| **Domain/items** | **Number of items** | ***N*** | **Cronbach’s α** | **Cronbach’s α if item deleted** | |
| --- | --- | --- | --- | --- | --- |
| Desire to Eat | 6 | 385 | 0.852 |  | |
| Item 1: Feeling emotional |  |  |  | 0.827 | |
| Item 2: Eating with family and friends |  |  |  | 0.853 | |
| Item 5: Indulge in comfort foods |  |  |  | 0.813 | |
| Item 6: When you were bored |  |  |  | 0.820 | |
| Item 7: Eat your favorite unhealthy foods |  |  |  | 0.818 | |
| Item 8: Eat foods readily available |  |  |  | 0.828 | |
| Capacity to Resist | 6 | 385 | 0.898 |  | |
| Item 11: Fight against eating habits |  |  |  | 0.880 | |
| Item 12: Make good food choices |  |  |  | 0.887 | |
| Item 13: Control portion size |  |  |  | 0.883 | |
| Item 14: Control eating when hungry |  |  |  | 0.882 | |
| Item 15: Avoid buying foods resist |  |  |  | 0.880 | |
| Item 16: Avoid eating foods resist |  |  |  | 0.867 | |
| Total Eating Behavior score | 12 | 385 | 0.926 |  | |
| Item 1: Feeling emotional |  |  |  | 0.923 | |
| Item 2: Eating with family and friends |  |  |  | 0.928 | |
| Item 5: Indulge in comfort foods |  |  |  | 0.920 | |
| Item 6: When you were bored |  |  |  | 0.920 | |
| Item 7: Eat your favorite unhealthy foods |  |  |  | 0.918 | |
| Item 8: Eat foods readily available |  |  |  | 0.920 | |
| Item 11: Fight against eating habits |  |  |  | 0.918 | |
| Item 12: Make good food choices |  |  |  | 0.921 | |
| Item 13: Control portion size |  |  |  | 0.920 | |
| Item 14: Control eating when hungry |  |  |  | 0.919 | |
| Item 15: Avoid buying foods resist |  |  |  | 0.919 | |
| Item 16: Avoid eating foods resist |  |  |  | 0.916 | |
| Abbreviation: EB PRO, Eating Behavior Patient-Reported Outcome. | | | | |  |

#### **Table S7** Test-test reliability of the EB PRO measure during screening^a^

| **Time period** | ***N*^b^** | **Score in time period**  **mean (SD)** | **Score at week 0**  **mean (SD)** | **Score difference**  **mean** | ***t* value** | ***p* value** | **ICC** |
| --- | --- | --- | --- | --- | --- | --- | --- |
| Week –12 to –1 (screening) to week 0 (baseline) |  |  |  |  |  |  |  |
| Desire to Eat | 218 | 11.07 (4.80) | 10.65 (4.96) | 0.43 | 1.93 | 0.0548 | 0.77 |
| Capacity to Resist | 219 | 11.05 (4.91) | 10.65 (5.21) | 0.40 | 1.63 | 0.1039 | 0.74 |
| Eating Habits (Homeostatic)^c^ | 219 | 10.63 (3.32) | 10.17 (3.32) | 0.46 | 2.97 | 0.0034 | 0.75 |
| Total Eating Behavior score^d^ | 219 | 22.07 (9.10) | 21.29 (9.62) | 0.78 | 2.00 | 0.0471 | 0.81 |
| Week –4 to –1 (screening) to week 0 (baseline) |  |  |  |  |  |  |  |
| Desire to Eat | 127 | 11.17 (4.51) | 10.39 (5.13) | 0.77 | 2.79 | 0.0062 | 0.78 |
| Capacity to Resist | 128 | 10.71 (4.80) | 10.01 (5.18) | 0.70 | 2.23 | 0.0275 | 0.74 |
| Eating Habits (Homeostatic)^c^ | 128 | 10.58 (3.52) | 10.22 (3.51) | 0.36 | 1.71 | 0.0905 | 0.77 |
| Total Eating Behavior score^d^ | 128 | 21.79 (8.74) | 20.40 (9.76) | 1.39 | 2.80 | 0.0059 | 0.81 |
| Week –2 to –1 (screening) to week 0 (baseline) |  |  |  |  |  |  |  |
| Desire to Eat | 39 | 10.51 (4.23) | 9.15 (4.80) | 1.36 | 2.88 | 0.0066 | 0.76 |
| Capacity to Resist | 39 | 10.23 (5.08) | 9.10 (4.91) | 1.13 | 2.16 | 0.0372 | 0.77 |
| Eating Habits (Homeostatic)^c^ | 39 | 10.77 (2.84) | 9.74 (3.38) | 1.03 | 2.55 | 0.0148 | 0.65 |
| Total Eating Behavior score^d^ | 39 | 20.74 (8.67) | 18.26 (9.03) | 2.49 | 2.96 | 0.0053 | 0.80 |
| *Note*: *p* values are for paired *t* test.  Abbreviations: EB PRO, Eating Behavior Patient-Reported Outcome; ICC, intraclass correlation coefficient; PGIS, Patient Global Impression of Severity; SD, standard deviation.  ^a^In the clinical trial in which the psychometric evaluation was conducted, there was a screening period of a minimum of one week that could be extended up to 12 weeks as needed to check eligibility criteria; these analyses were for either participants completing the EB PRO measure any time during this period before baseline (when the EB PRO was administered again), or those completing it within four weeks of baseline, or those completing it within two weeks of baseline.  ^b^Data are for participants with no change during the time period in the PGIS for Eating Behavior (*Overall, how would you rate your eating behavior over the last 7 days*).  ^c^Data from the draft measure.  ^d^Total Eating Behavior score is for the final EB PRO measure including only the Desire to Eat and Capacity to Resist domains. | | | | | | | |

#### **Table S8** Baseline daily energy and macronutrient intake based on three-day food diaries

| **Treatment** | **n** | **Energy**  **(kcal)** | **Carbohydrates**  **(%)** | **Fat**  **(%)** | **Protein**  **(%)** |
| --- | --- | --- | --- | --- | --- |
| Survodutide 0.6 mg | 74 | 1979.3 (906.6) | 43.3 (15.2) | 33.5 (10.8) | 18.1 (5.9) |
| Survodutide 2.4 mg | 69 | 1779.9 (547.8) | 41.6 (11.6) | 32.6 (11.2) | 21.1 (7.8) |
| Survodutide 3.6 mg | 69 | 1850.6 (635.1) | 42.3 (12.7) | 32.9 (11.6) | 20.1 (9.2) |
| Survodutide 4.8 mg | 72 | 1730.6 (652.2) | 41.9 (11.7) | 33.1 (10.3) | 21.1 (8.9) |
| Placebo | 70 | 1869.8 (608.7) | 46.4 (12.1) | 33.9 (10.8) | 19.7 (8.1) |
| Data are mean (standard deviation). | | | | | |

#### **Table S9** Known-groups validity: EB PRO score by PGIS

| **EB PRO** | **PGIS scale** | | | | | ***F* value (*p* value)** |
| --- | --- | --- | --- | --- | --- | --- |
|  | **Extremely**  ***n*, mean (SD)** | **Very**  ***n*, mean (SD)** | **Somewhat**  ***n*, mean (SD)** | **A little**  ***n*, mean (SD)** | **Not at all**  ***n*, mean (SD)** |  |
| Baseline |  |  |  |  |  |  |
| Desire to Eat | 70, 14.77 (4.11) | 171, 11.32 (4.31) | 120, 8.65 (4.24) | 23, 8.57 (4.98) | 1, 6.00 (n/a) | 24.86 (<0.0001) |
| Capacity to Resist | 70, 15.20 (4.28) | 171, 11.71 (4.55) | 120, 8.22 (4.37) | 23, 7.65 (4.95) | 1, 3.00 (n/a) | 32.19 (<0.0001) |
| Total Eating Behavior score | 70, 29.97 (7.58) | 171, 23.03 (8.17) | 120, 16.87 (8.13) | 23, 16.22 (9.72) | 1, 9.00 (n/a) | 32.83 (<0.0001) |
| Week 20 |  |  |  |  |  |  |
| Desire to Eat | 15, 11.47 (7.31) | 59, 7.83 (3.95) | 118, 6.01 (3.62) | 82, 4.73 (2.91) | 13, 4.15 (5.83) | 13.33 (<0.0001) |
| Capacity to Resist | 15, 11.60 (7.32) | 59, 7.15 (3.97) | 118, 5.36 (3.66) | 82, 3.24 (2.81) | 13, 3.00 (5.29) | 20.35 (<0.0001) |
| Total Eating Behavior score | 15, 23.07 (13.89) | 59, 14.98 (7.33) | 118, 11.36 (6.75) | 82, 7.98 (5.11) | 13, 7.15 (10.73) | 19.19 (<0.0001) |
| Week 46 |  |  |  |  |  |  |
| Desire to Eat | 20, 11.25 (5.32) | 92, 9.04 (3.77) | 125, 6.34 (3.74) | 85, 4.68 (3.34) | 25, 2.48 (2.37) | 31.82 (<0.0001) |
| Capacity to Resist | 20, 12.70 (5.80) | 92, 8.29 (4.22) | 125, 5.48 (3.78) | 85, 3.19 (2.87) | 25, 1.60 (1.89) | 45.43 (<0.0001) |
| Total Eating Behavior score | 20, 23.95 (10.84) | 92, 17.34 (7.40) | 125, 11.82 (6.92) | 85, 7.87 (5.55) | 25, 4.08 (3.77) | 44.78 (<0.0001) |
| *Note*: *F* and *p* values from ANOVA.  Abbreviations: ANOVA, analysis of variance; EB PRO, Eating Behavior Patient-Reported Outcome measure; n/a, not applicable (SD not calculable as *n* = 1); PGIS, Patient Global Impression of Severity (of hunger over previous 7 days); SD, standard deviation. | | | | | | |

#### **Table S10** Known-groups validity: EB PRO score by BMI

| **EB PRO** | **BMI category (kg/m^2^)** | | | | ***F* value (*p* value)** |
| --- | --- | --- | --- | --- | --- |
|  | **25 to <30**  ***n*, mean (SD)** | **30 to <35**  ***n*, mean (SD)** | **35 to <40**  ***n*, mean (SD)** | **≥40**  ***n*, mean (SD)** |  |
| Baseline |  |  |  |  |  |
| Desire to Eat | 40, 9.25 (4.91) | 117, 10.96 (4.83) | 121, 11.21 (4.55) | 107, 11.23 (4.94) | 1.92 (0.1250) |
| Capacity to Resist | 40, 8.88 (5.33) | 117, 11.24 (5.43) | 121, 11.38 (4.91) | 107, 11.07 (4.90) | 2.62 (0.0507) |
| Total Eating Behavior score | 40, 18.13 (9.86) | 117, 22.20 (9.74) | 121, 22.59 (8.82) | 107, 22.31 (9.31) | 2.50 (0.0595) |
| Week 20 |  |  |  |  |  |
| Desire to Eat | 74, 5.20 (3.20) | 107, 5.85 (4.28) | 57, 6.95 (4.48) | 41, 8.41 (4.44) | 6.33 (0.0004) |
| Capacity to Resist | 74, 3.86 (2.89) | 107, 5.25 (4.64) | 57, 6.30 (4.36) | 41, 7.17 (4.95) | 6.51 (0.0003) |
| Total Eating Behavior score | 74, 9.07 (5.45) | 107, 11.10 (8.48) | 57, 13.25 (8.41) | 41, 15.59 (8.72) | 7.13 (0.0001) |
| Week 46 |  |  |  |  |  |
| Desire to Eat | 86, 5.21 (4.18) | 107, 6.41 (4.09) | 65, 8.43 (4.47) | 56, 7.98 (4.00) | 9.24 (<0.0001) |
| Capacity to Resist | 86, 4.43 (3.99) | 107, 5.64 (4.60) | 65, 7.65 (4.73) | 56, 7.36 (5.08) | 8.08 (<0.0001) |
| Total Eating Behavior score | 86, 9.64 (7.62) | 107, 12.06 (8.16) | 65, 16.08 (8.79) | 56, 15.34 (8.66) | 9.70 (<0.0001) |
| *Note*: *F* and *p* values from ANOVA.  Abbreviations: ANOVA, analysis of variance; BMI, body mass index; EB PRO, Eating Behavior Patient-Reported Outcome measure; SD, standard deviation. | | | | | |

#### **Table S11** Known-groups validity: EB PRO score by PGIC

| **EB PRO** | **PGIC scale** | | | | | ***F* value (*p* value)** |
| --- | --- | --- | --- | --- | --- | --- |
|  | **Much better**  ***n*, mean (SD)** | **A little better**  ***n*, mean (SD)** | **No change**  ***n*, mean (SD)** | **A little worse**  ***n*, mean (SD)** | **Much worse**  ***n*, mean (SD)** |  |
| Week 20 |  |  |  |  |  |  |
| Desire to Eat | 176, 4.93 (3.16) | 94, 8.17 (4.90) | 12, 8.25 (5.07) | 5, 9.80 (4.09) | 0 | 16.71 (<0.0001) |
| Capacity to Resist | 176, 3.99 (3.29) | 94, 7.44 (4.96) | 12, 7.50 (5.84) | 5, 8.00 (3.32) | 0 | 16.95 (<0.0001) |
| Total Eating Behavior score | 176, 8.91 (5.90) | 94, 15.61 (9.30) | 12, 15.75 (10.45) | 5, 17.80 (6.30) | 0 | 19.35 (<0.0001) |
| Week 46 |  |  |  |  |  |  |
| Desire to Eat | 185, 5.11 (3.58) | 111, 8.33 (3.89) | 36, 8.81 (5.59) | 10, 8.40 (4.01) | 5, 7.80 (5.89) | 15.17 (<0.0001) |
| Capacity to Resist | 185, 4.03 (3.52) | 111, 7.61 (4.17) | 36, 8.14 (6.25) | 10, 9.50 (6.42) | 5, 6.80 (6.42) | 17.99 (<0.0001) |
| Total Eating Behavior score | 185, 9.14 (6.46) | 111, 15.95 (7.56) | 36, 16.94 (11.51) | 10, 17.90 (10.03) | 5, 14.60 (12.16) | 18.86 (<0.0001) |
| *Note*: *F* and *p* values from ANOVA.  Abbreviations: ANOVA, analysis of variance; EB PRO, Eating Behavior Patient-Reported Outcome measure; PGIC, Patient Global Impression of Change (of eating behavior since study start); SD, standard deviation. | | | | | | |

#### **Table S12** Change-score correlations of the EB PRO and PGIS and PGIC

| **EB PRO change score** | **PCIS** | | | | | | **PGIC score** |
| --- | --- | --- | --- | --- | --- | --- | --- |
|  | **Desire to eat**  **change score** | **Food cravings**  **change score** | **Resisting food cravings**  **change score** | **Severity of hunger**  **change score** | **Having healthy eating behavior**  **change score** | **Overall eating behavior**  **change score** |  |
| Desire to Eat score | 0.50*** | 0.54*** | –0.21*** | 0.48*** | 0.38*** | 0.36*** | 0.33*** |
| Capacity to Resist score | 0.48*** | 0.53*** | –0.25*** | 0.46*** | 0.47*** | 0.39*** | 0.29*** |
| Total Eating Behavior score | 0.53*** | 0.58*** | –0.25*** | 0.51*** | 0.47*** | 0.42*** | 0.33*** |
| *Note*: Data are Spearman’s rank correlation coefficients for changes from baseline to week 46.  Abbreviations: EB PRO, Eating Behavior Patient-Reported Outcome; PGIC, Patient Global Impression of Change; PGIS, Patient Global Impression of Severity.  **p* < 0.05; ***p* < 0.001; ****p* < 0.0001. | | | | | | | |

**Figure S1** Anchor-based analysis of meaningful change threshold by (A) PGIS score and (B) PGIC score. EB PRO, Eating Behavior Patient-Reported Outcome measure; PGIC, Patient Global Impression of Change; PGIS, Patient Global Impression of Severity; SE, standard error.

**
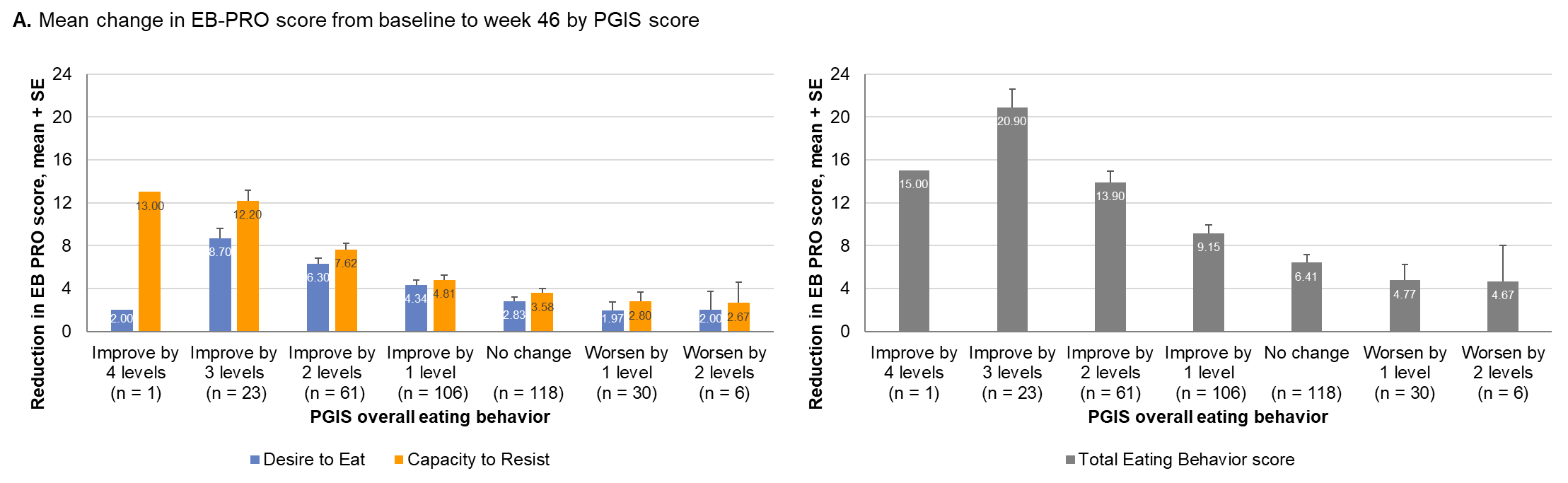

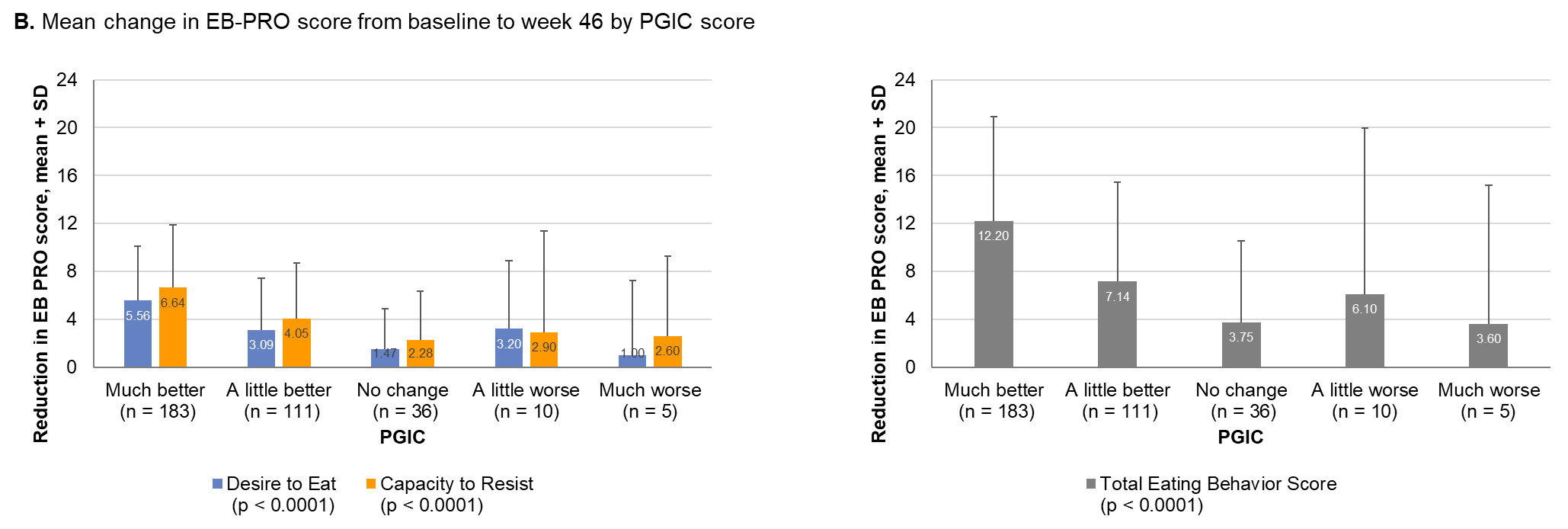
**
